# Intestinal NADPH Oxidase 2 Activity Increases in a Neonatal Rat Model of Necrotizing Enterocolitis

**DOI:** 10.1371/journal.pone.0115317

**Published:** 2014-12-17

**Authors:** Scott R. Welak, Rebecca M. Rentea, Ru-Jeng Teng, Nathan Heinzerling, Ben Biesterveld, Jennifer L. Liedel, Kirkwood A. Pritchard, Katherine M. Fredrich, David M. Gourlay

**Affiliations:** 1 Division of Neonatology, Department of Pediatrics, Medical College of Wisconsin, Milwaukee, Wisconsin, United States of America; 2 Children's Research Institute, Medical College of Wisconsin, Milwaukee, Wisconsin, United States of America; 3 Translational Vascular Biology Program, Medical College of Wisconsin, Milwaukee, Wisconsin, United States of America; 4 Division of Pediatric Surgery, Department of Surgery, Medical College of Wisconsin, Milwaukee, Wisconsin, United States of America; 5 Division of Critical Care, Department of Pediatrics, Medical College of Wisconsin, Milwaukee, Wisconsin, United States of America; Goethe Universităt Frankfurt, Germany

## Abstract

Necrotizing enterocolitis (NEC) is a complication of prematurity. The etiology is unknown, but is related to enteral feeding, ischemia, infection, and inflammation. Reactive oxygen species production, most notably superoxide, increases in NEC. NADPH oxidase (NOX) generates superoxide, but its activity in NEC remains unknown. We hypothesize that NOX-derived superoxide production increases in NEC. Newborn Sprague-Dawley rats were divided into control, formula-fed, formula/LPS, formula/hypoxia, and NEC (formula, hypoxia, and LPS). Intestinal homogenates were analyzed for NADPH-dependent superoxide production. Changes in superoxide levels on days 0-4 were measured. Inhibitors for nitric oxide synthase (L-NAME) and NOX2 (GP91-ds-*tat*) were utilized. RT-PCR for eNOS, NOX1, GP91^phox^ expression was performed. Immunofluorescence studies estimated the co-localization of p47^phox^ and GP91^phox^ in control and NEC animals on D1, D2, and D4. NEC pups generated more superoxide than controls on D4, while all other groups were unchanged. NADPH-dependent superoxide production was greater in NEC on days 0, 3, and 4. GP91-ds-*tat* decreased superoxide production in both groups, with greater inhibition in NEC. L-NAME did not alter superoxide production. Temporally, superoxide production varied minimally in controls. In NEC, superoxide generation was decreased on day 1, but increased on days 3-4. GP91^phox^ expression was higher in NEC on days 2 and 4. NOX1 and eNOS expression were unchanged from controls. GP91^phox^ and p47^phox^ had minimal co-localization in all control samples and NEC samples on D1 and D2, but had increased co-localization on D4. In conclusion, this study proves that experimentally-induced NEC increases small intestinal NOX activity. All components of NEC model are necessary for increased NOX activity. NOX2 is the major source, especially as the disease progresses.

## Introduction

Necrotizing enterocolitis (NEC) is one of the most devastating diseases for premature infants. The disease involves injury and death of the small intestines. NEC causes significant neonatal morbidity and mortality, and survivors encounter many long-term sequelae [1]. Despite its high prevalence, and years of investigation, the pathogenesis remains unclear. NEC is thought to be a multi-factorial disease process [2]. Enteral feeding, infection, and intestinal ischemia play vital roles in the pathogenesis of NEC [2]. Vascular dysfunction and inflammation may also contribute to the disease [3].

One factor postulated to play a key role in NEC pathogenesis is oxidative stress [3]. Superoxide (O_2_^•^–) is a potent source of oxidative stress. Four enzymatic sources of O_2_^•^– exist: mitochondria, xanthine oxidase/dehydrogenase, uncoupled nitric oxide synthase (NOS), and NADPH oxidase (NOX). Two of these pathways, NOS and NOX, require NADPH as a cofactor. Under homeostatic conditions, NOS generates nitric oxide, but in times of cellular injury, NOS can become uncoupled to generate O_2_^•^– [4]. Previously, this laboratory showed that uncoupled endothelial NOS (eNOS) activity produces increased O_2_^•^– in neonatal rat mesenteric arteries in experimentally-induced NEC [5]. However, O_2_^•^– production in the small intestines has not been thoroughly examined.

There are several NOX isoforms, which generate either O_2_^•^– or hydrogen peroxide (H_2_O_2_) [6]. NOX enzymes contribute to oxidative stress in both physiologic and pathologic states. The first two isoforms (NOX1 and NOX2) generate O_2_^•^–. NOX2, found predominantly in neutrophils and macrophages, is the prototypical NOX isoform, and contributes to the phagocytic respiratory burst in host defense [6]. Absent NOX2 subunits results in the immunodeficiency disorder known as chronic granulomatous disease [7]. Conversely, excess O_2_^•^– production from NOX2 occurs in many pathologic states, including septic shock, cardiovascular disease, and diabetes [6], [8]–[12].

NOX1 also generates O_2_^•^–, but has a more diffuse distribution [6]. The physiologic functions of NOX1 are incompletely understood. Vascular remodeling and angiogenesis are associated with NOX1 [13], [14]. As with NOX2, elevated O_2_^•^– production from NOX1 may contribute to inflammation and injury. Elevated NOX1 activity is associated with vascular injury [15] and preeclampsia [16]. In addition, gastrointestinal diseases such as small intestinal mucositis [17], inflammatory bowel disease [18], and colon cancer [19] have been associated with increased NOX1 levels.

Inflammatory processes often involve leukocyte activation. Previously, leukocytes have been shown to be mediators of injury in experimental NEC animal models [20]. The etiology for the injury is not well understood. Because O_2_^•^– generated by NOX2 increases in leukocyte activation, the association between O_2_^•^– levels and NEC needs further understanding.

The contribution of NOX to the overall O_2_^•^– generation in NEC and its relevance to it pathogenesis has not been studied. The Sprague Dawley newborn rat model of NEC has been extensively utilized in basic science research for NEC, and provides a comparable phenotype to that observed clinically. We hypothesize that the generation of O_2_^•^– by NOX1 and NOX2 play crucial roles underlying the inflammation of NEC. In this study, we examine how experimentally-induced NEC is associated with NOX-dependent O_2_^•^– production. In addition, the contribution of specific NOX isoforms will be delineated.

## Results

### Experimentally-induced NEC is associated with increased NAPDH-dependent O_2_^•^– production

In order to confirm that small intestinal O_2_^•^– generation is via NOX and NOS, samples were analyzed in the presence or absence of NADPH as described above. When NADPH was withheld from the reaction, there was an absence of chemiluminescence in every sample.

**Figure 1:**
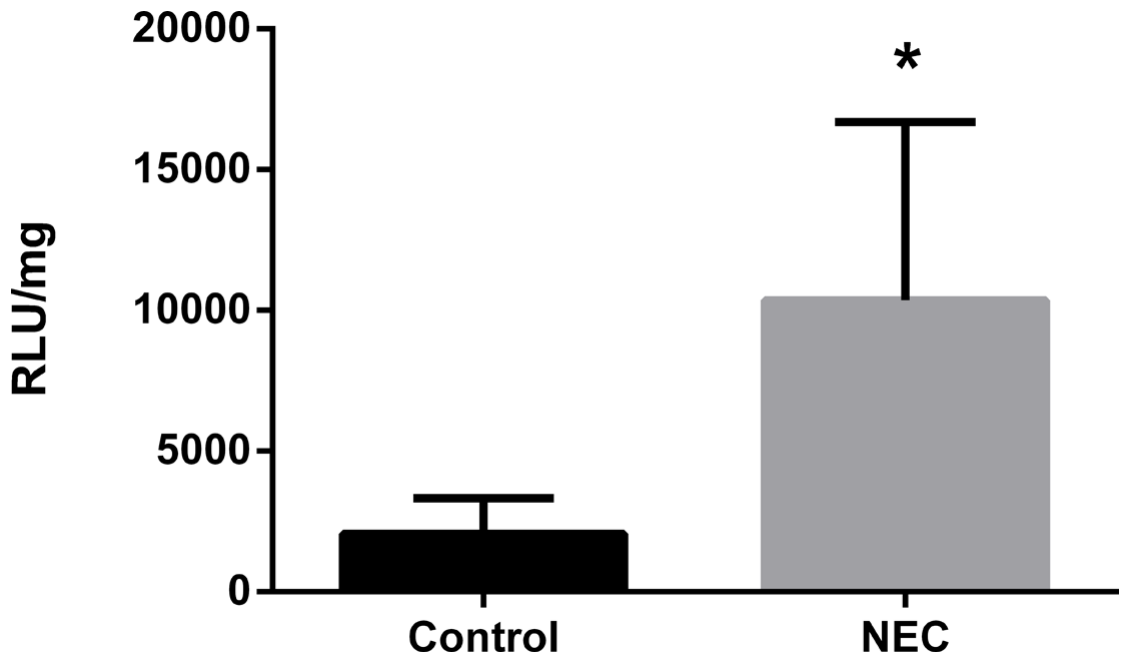
NADPH-dependent O_2_^•^– production in small intestinal homogenates from control (n = 6) and NEC (n = 5) on D4. *  =  p<0.05.

After confirmation that O_2_^•^– generation is NADPH dependent, control and NEC samples from D4 were analyzed (Fig. 1). There was a three-fold increase in O_2_^•^– production in NEC animals compared to controls (p<0.05).

### LPS, Hypoxia, and Formula are required for increased O_2_^•^– generation

**Figure 2:**
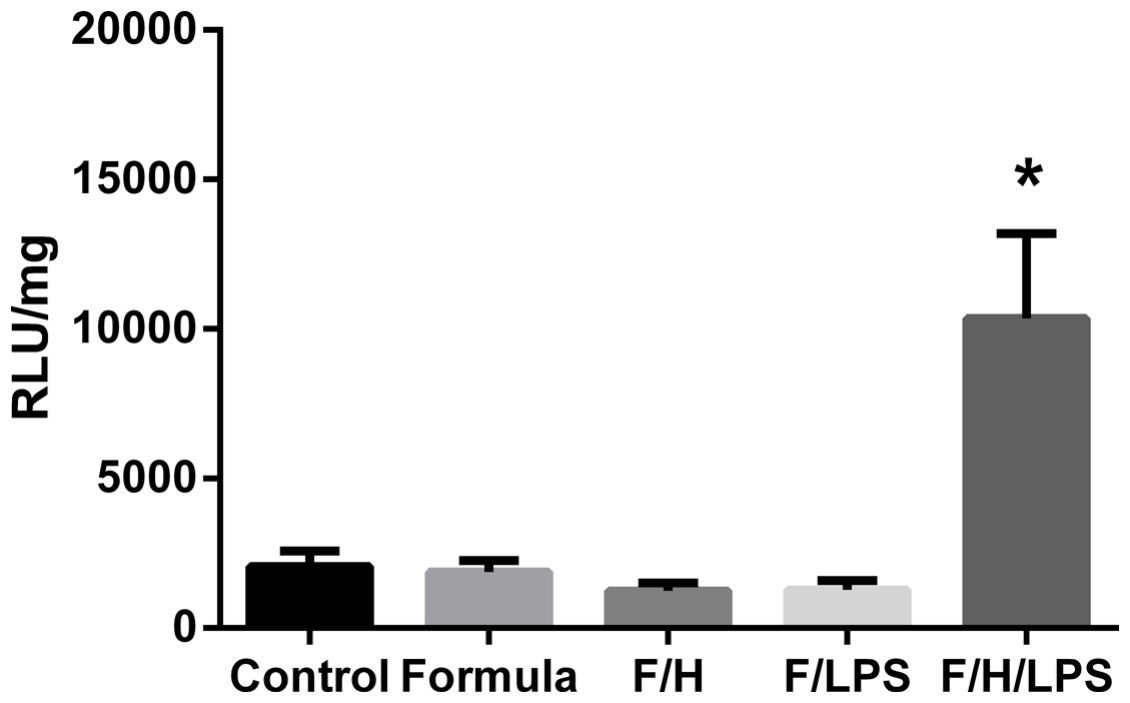
NADPH-dependent O_2_^•^– production in small intestinal homogenates from D4. n = 5–7 per group F =  Formula, F/H  =  Formula and hypoxia, F/L  =  Formula and LPS, F/H/L  =  Formula, Hypoxia, and LPS (NEC model). *  =  p<0.05 between NEC and each group.

Because pups exposed to all three components of NEC had increased O_2_^•^– generation, it was important to determine the contribution of each component in causing elevated O_2_^•^– production. Accordingly, the study was repeated with pups received formula (F), formula and hypoxia (F/H), and formula and LPS (F/LPS) (Fig. 2). Pups who received F, F/H, or F/LPS did not have increased NOX activity compared to control pups (*p* =  NS by ANOVA). However, NEC pups had increased activity compared to pups in any other condition (p<0.05 for each group compared to NEC or ANOVA *p*<0.0001 with NEC group).

### Production of O_2_^•^– exhibits temporal differences

**Figure 3:**
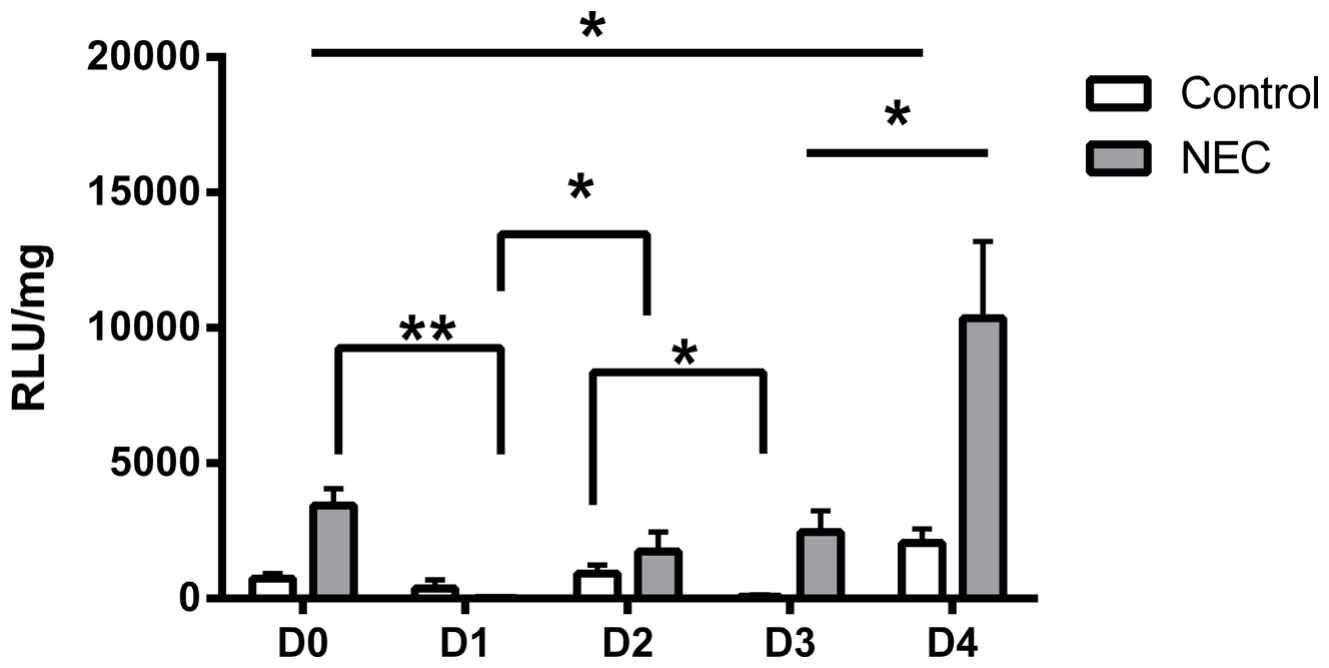
NADPH-dependent O_2_^•^– production in small intestinal homogenates from D0-4. Same-condition experimental groups that had significant differences represented by error bars. Both control and NEC samples were significantly different between D0 and D4. In addition, significant differences between control and NEC samples occurred on D0, D3, and D4 (p<0.05). n = 5–6 per group *  =  p<0.05, ** p<0.01.

After confirmation that all components of the NEC model were required for maximal NOX activity, the assay was used to quantify O_2_^•^– production over the first 5 days of life (Fig. 3). In control rats, there were no significant changes in O_2_^•^–production from day of life 0 (D0) to D1, nor D1 to D2. Pups from D3 were significantly decreased compared to D2 (*p*<0.05). From D3 to D4, there was another significant increase (*p*<0.01). Comparing D0 to D4, there was nearly a three-fold increase NOX activity (*p*<0.05).

NEC pups also exhibited changes during the study. There was a significant decrease from D0 to D1 (*p*<0.005). On D2, O_2_^•^– production significantly increased over D1 (*p*<0.05). D3 samples exhibited a non-significant increase from D2. A four-fold increase, the largest in the study, occurred on D4 (*p*<0.01). From D0 to D4, there was also a four-fold increase in activity (p<0.05).

In addition to changes observed within each condition, control and NEC pups exhibited different activities on the same day of the study. NEC pups had significantly elevated O_2_^•^– generation activity when compared to controls on D0, D3, and D4 (*p*<0.05).

### NOX2, but not NOS, is a significant source of O_2_^•^– generation

**Figure 4:**
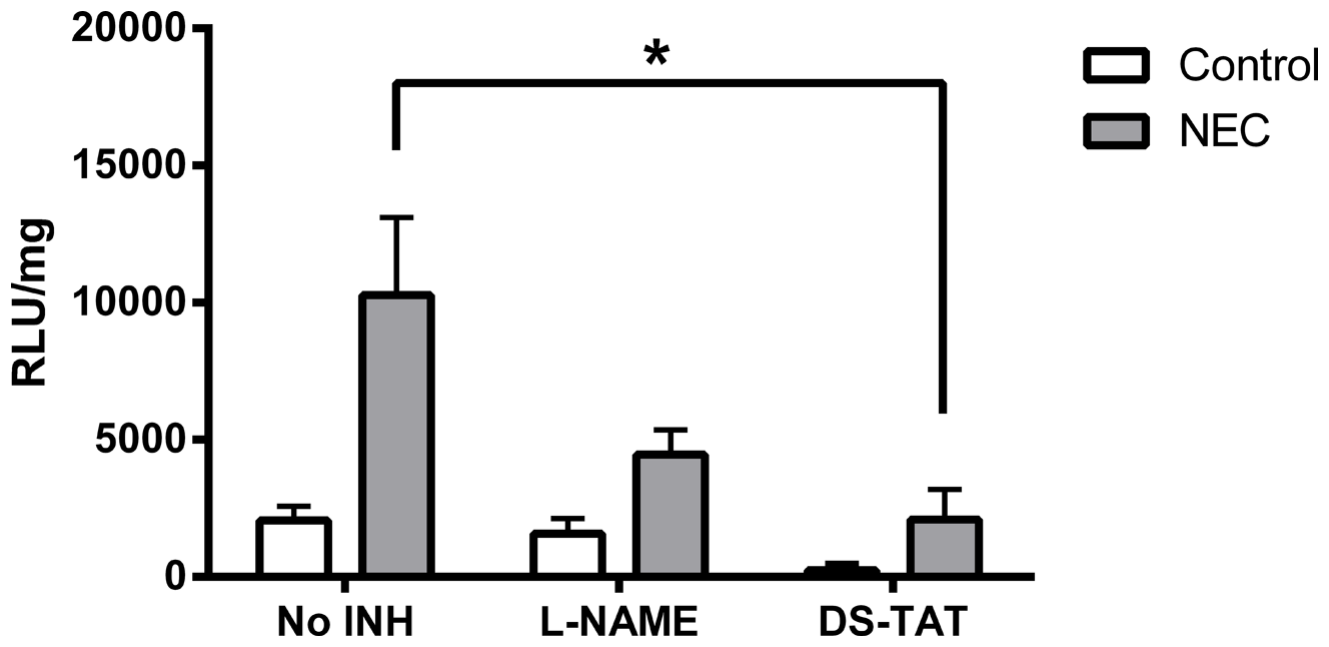
Effects of inhibitors on NADPH- dependent O_2_^•^– production on D4 small intestinal homogenates. n = 6 for control, 5 for NEC. *  =  p<0.05

Because NOS and NOX can generate O_2_^•^– and require NADPH, additional studies were needed to elucidate specific enzymatic contribution. The chemiluminescent assay was performed on tissue homogenates in the presence of specific inhibitors for NOS (*N*_ω_-Nitro-L-arginine methyl ester hydrochloride, L-NAME) and NOX2 (GP91-ds-*tat*). Control and NEC samples from D4 were exposed to inhibitors, and O_2_^•^– production quantified (Fig. 4). Although L-NAME tended to reduce O_2_^•^– production in both control and NEC samples, the differences did not achieve significance (p>0.05).

In contrast, GP91-ds-*tat* reduced O_2_^•^– production in both control and NEC samples by nearly 90% (*p* = 0.01). Importantly, samples from NEC pups treated with GP91-ds-*tat* generated O_2_^•^– at essentially the same levels as to untreated control samples.

### GP91^phox^ expression is increased in NEC

To further examine specific enzyme contributions, RT-PCR was performed for GP91^phox^ (NOX2 catalytic site), NOX1, and eNOS. Results were normalized to expression observed on D0 from control pups.

**Figure 5:**
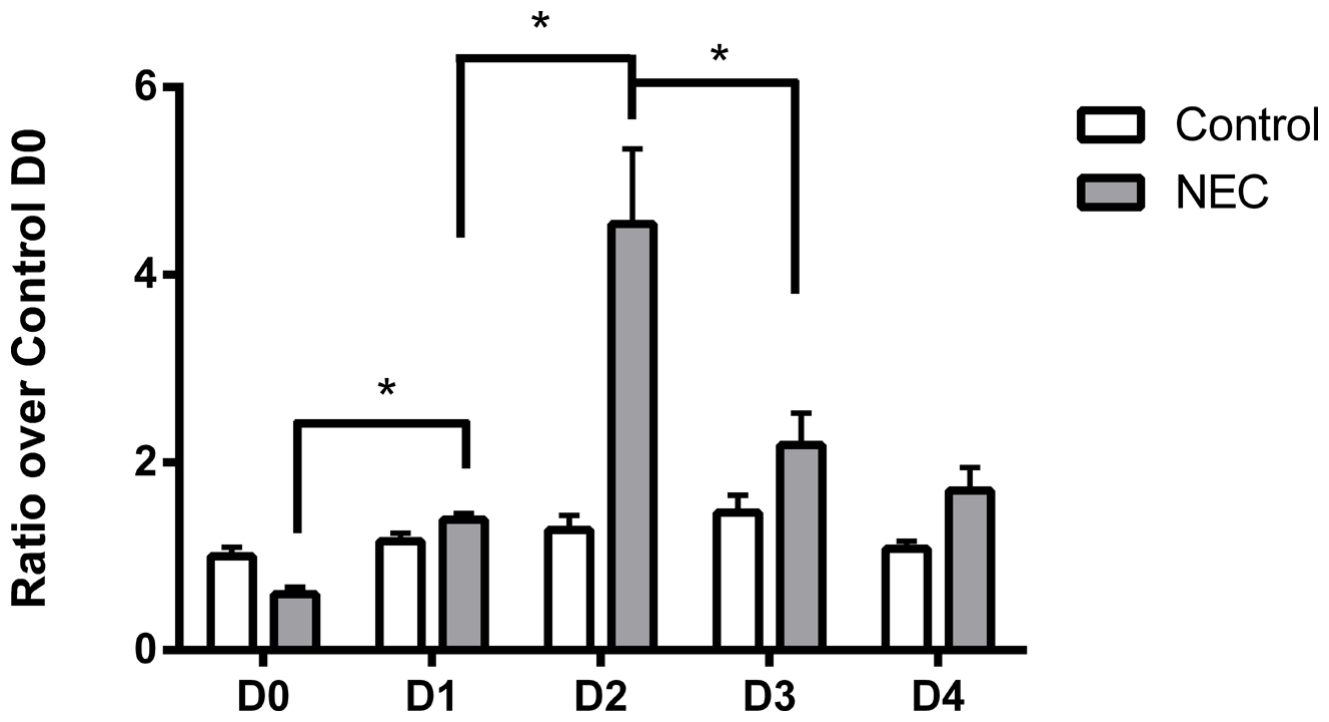
RT-PCR results for GP91^phox^. Same day differences between control and NEC occurred on D0, D2, and D4. n = 6 per group. *  =  p<0.005.

GP91^phox^ expression in control pups exhibited minimal variation among the five days studied (Fig. 5). Differences observed were not significant (*p*>0.1 by ANOVA). The highest expression occurred on D3 (1.47±0.18 fold increase over D0). There were no significant differences observed between days.

While GP91^phox^ expression in control pups remained constant, NEC pups exhibited varying levels of expression. From D0 to D1, expression increased (0.6±0.08 to 1.39±0.06 fold, p = 0.0001). From D1 to D2, there was a three-fold difference in expression (1.39±0.06 to 4.54±0.8 fold, p<0.005). Expression significantly decreased on D3 (2.19±0.34, p<0.05), followed by a non-significant decrease on D4 (1.7±0.25, p>0.05).

Similar to the chemiluminescent study, same-day differences were observed between groups. Expression was significantly decreased in NEC pups on D0 (1±0.096 vs. 0.6±0.08 fold, *p*<0.01). On D2, expression in NEC pups was three-fold greater than controls (4.54±0.8 vs. 1.28±0.16, *p*<0.005). Expression in NEC animals was also significantly increased compared to control samples on D4 (1.70±0.25 vs. 1.08±0.08 fold, *p*<0.05). There were no differences on D1 and D3.

### Expressions of NOX1 and eNOS do not increase in NEC samples

**Figure 6:**
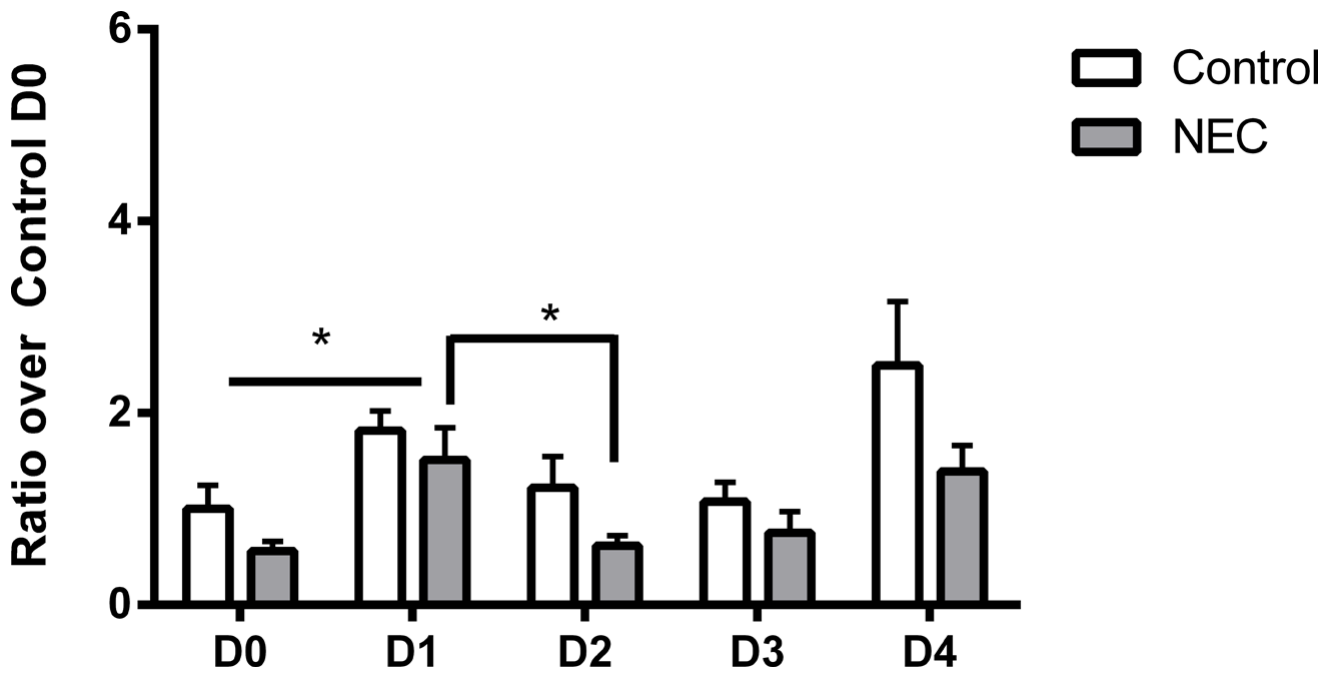
RT-PCR results for NOX1. Both control and NEC samples increased from D0 to D1. There were no same-day differences between control and NEC. n = 4–5 per group. *  =  p<0.05.

Because NOX1 also can generate O_2_^•^–, RT-PCR for this isoform was measured (Fig. 6). Both control and NEC samples exhibited significant differences during the study (*p*<0.05 by ANOVA for each condition). However, different trends were observed in the two conditions.

Control samples had increased expression between D0 and D1 (1.00±0.25 vs. 1.82±0.21 fold, *p*<0.05). There were no significant differences between D1 and D2 (1.22±0.33), and D2 and D3 (1.08±0.2 fold). NOX1 expression in controls tended to increase on D4 (2.5±0.66), but the differences did not achieve statistical significance (*p*>0.05). The differences observed from D0 to D4 were also not significant (1±0.25 vs. 2.5±0.66 fold, *p*>0.05).

NEC pups had different expression patterns in NOX1. Expression increased from D0 to D1 (0.56±0.099 vs. 1.51±0.34, *p*<0.05), followed by a decrease from D1 to D2 (0.62±0.11, *p*<0.05). While NOX1 expression increased on both D3 (0.75±0.22 fold) and D4 (1.39±0.27 fold), neither achieved statistical significance (p>0.05).

NOX1 expression was higher in controls compared to NEC pups during every day throughout the study. Expression was higher in controls on D0 (1±0.25 vs. 0.56±0.099 fold, *p*<0.05), and D1 (1.82±0.21 vs. 1.51±0.34, *p*<0.05). There were no differences on D2-4.

**Figure 7:**
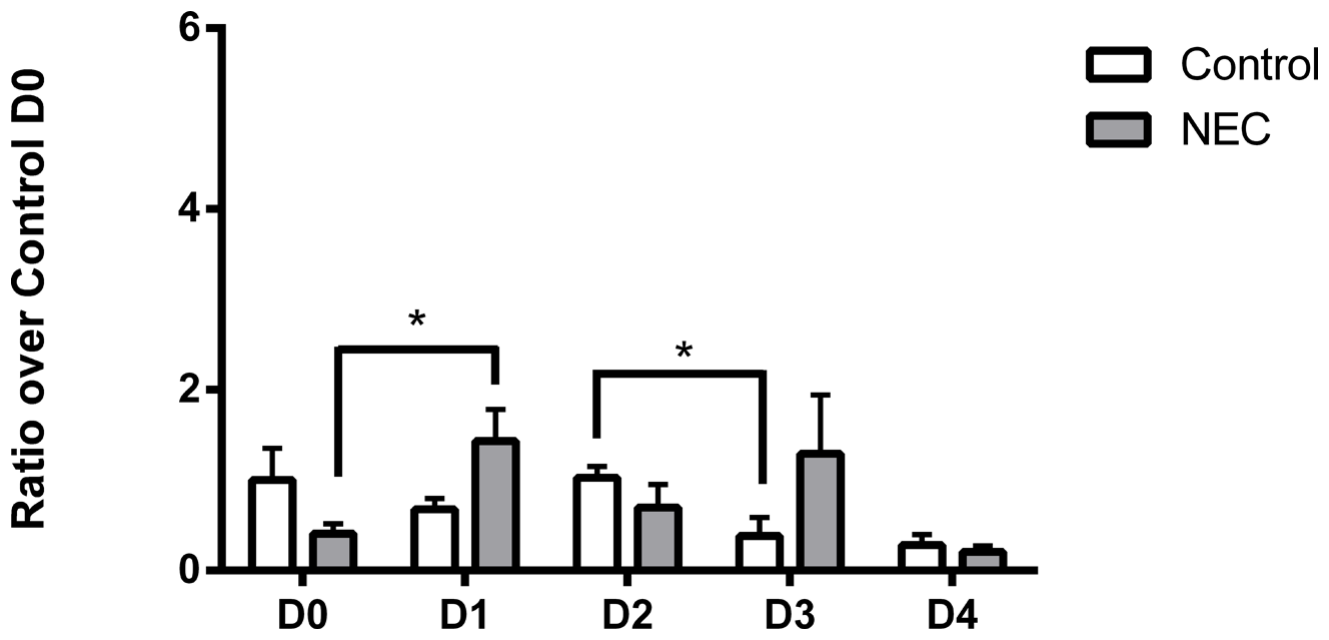
RT-PCR results for eNOS. There were no same-day differences between control and NEC. n = 4–6 per group. *  =  p<0.05.

Previous research from our laboratory identified uncoupled eNOS as a source of O_2_^•^– in NEC in isolated mesentery [5]. To determine if eNOS is involved, RT-PCR for eNOS was performed (Fig. 7). In contrast to GP91^phox^ and NOX1 expression, values for eNOS were not significantly different over the time course for either control or NEC samples (p>0.1 by ANOVA for each group). The only significant change in control samples was from D2 to D3 (1.03±0.13 fold vs. 0.43±0.24, *p*<0.05). For NEC samples, values achieved significance comparing D0 to D1 (0.41±0.11 vs. 1.43±0.35, *p*<0.05). When control and NEC animals are compared on the same day, there were no significant differences observed.

### NOX2 subunits co-localize on D4 of NEC

There are six subunits that comprise the NOX2 complex: two transmembrane proteins (GP91^phox^ and p22^phox^) and four cytosolic proteins (p40^phox^, p47^phox^, p67^phox^, and Rac). In the inactive state, the cytosolic proteins do not interact with the transmembrane proteins. When p47^phox^ is phosphorylated, the cytosolic proteins translocate to the GP91^phox^/p22^phox^ complex. This collection of proteins is now capable of generating superoxide by reducing molecular oxygen with NADPH as a cofactor [21]. It is possible to indirectly assess the degree of NOX activity with studies that examine how specific subunits co-localize. Previous studies have demonstrated that co-localization of p47^phox^ and GP91^phox^ can be observed with immunofluorescence [22], [23]. To add evidence that neonatal pups have increased NOX2 activity after intestinal injury in this NEC model, we performed immunofluorescence for p47^phox^ and GP91^phox^.

**Figure 8:**
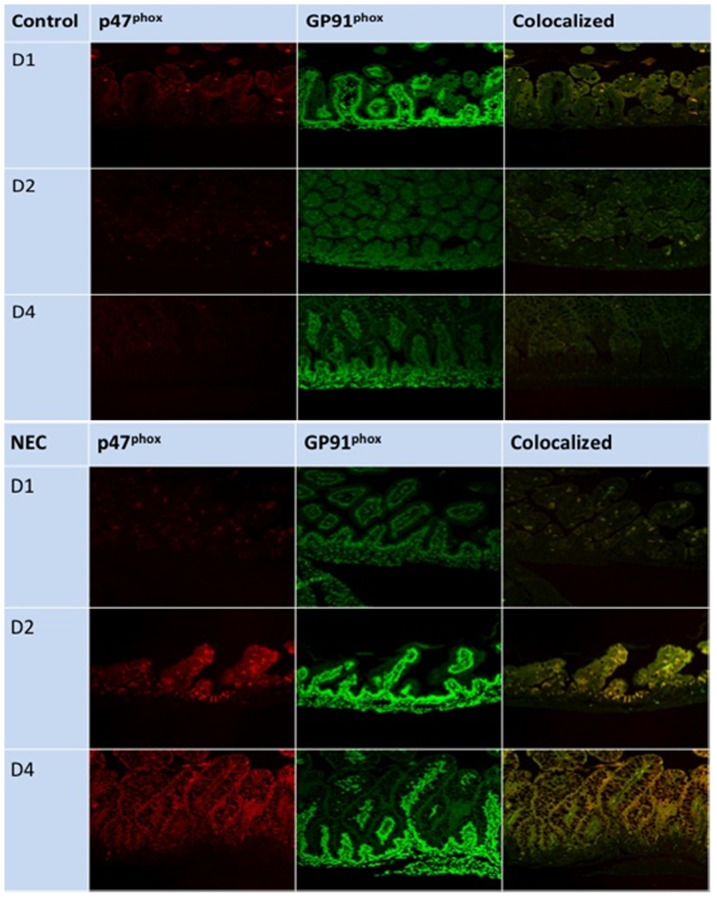
Immunofluorescence for p47^phox^ and GP91^phox^ on D1, D2, and D4. Fig. 8a shows control samples, and Fig. 8b shows NEC samples. The images are of p47^phox^ (red) GP91^phox^ (green), and the co-localization of the two proteins (yellow).

Representative samples from D1, D2, and D4 are seen in Fig. 8. The terminal ileum was evaluated by immunofluorescence for GP91^phox^ (green) and p47^phox^ (red), and for co-localization of the two proteins (yellow) as an index of activation. In the control terminal ileum (Fig. 8a), GP91^phox^ and p47^phox^ co-localization was minimal, and not increased compared to any of the control samples. In NEC terminal ileum (Fig. 8b), there was minimal co-localization of GP91^phox^ and p47^phox^ on D1 and D2. However, co-localization of GP91^phox^ and p47^phox^ on D4 was increased compared to all other samples.

## Discussion

NEC remains one of the most significant complications of extreme prematurity. Despite a high prevalence with serious morbidity and mortality, little improvement has been achieved towards understanding this disease. Inflammation and oxidative stress have been demonstrated to be integral to the development of NEC. Several studies have shown that premature infants with higher levels of oxidative stress markers are more likely to develop NEC [24]–[26]. In animal models of NEC, elevated levels of ROS have been implicated in the pathogenesis of NEC [27]–[30]. However, the enzymatic sources of increased ROS have not been thoroughly examined. NOX enzymes are significant sources of ROS in many diseases such as hypertension, diabetes, and atherosclerosis [31]. This study is the first to show that NOX-dependent O_2_^•^– production is increased in an animal model of NEC.

Several changes occurred in the NOX activity in this NEC model. There was nearly a five-fold difference in NOX activity in pups exposed to the combination of formula, hypoxia, and LPS compared to breast fed pups. Our data also indicate that all three components of the NEC model (formula feeding, hypoxia, and enteral LPS) are required to cause elevated NOX activity. Subjecting pups to only one or two stressors did not change NOX activity. This is consistent with clinical NEC, which is considered a multifactorial disease.

Uncoupled NOS has the potential to generate O_2_^•^– and requires NADPH as a cofactor. We examined whether iNOS or eNOS could be responsible for the changes observed. Expression of eNOS did not vary significantly in either control or NEC pups during the course of the study. We did not measure iNOS mRNA, as it has been shown to increase in experimental NEC [32]–[34]. L-NAME inhibits all forms of NOS [35], [36]. When L-NAME was included in the reaction, O_2_^•^– production was unchanged, indicating that both eNOS and iNOS are not significant sources of O_2_^•^– in this model.

Based on RT-PCR and inhibitor studies, NOX2 appears to be the most significant isoform responsible for changes in O_2_^•^– production. NOX2 mRNA expression increased dramatically on D2, compared to both same-day control and previous day NEC samples. This changed preceded the largest increase in O_2_^•^– production. Conversely NOX1 mRNA did not change as dramatically. At every time point, NOX1 mRNA levels were higher in control samples when compared to NEC samples, and expression decreased at the same time as O_2_^•^– generation rose. Additionally, O_2_^•^– production was decreased by 90% in the presence of GP91-ds-*tat* in both control and NEC pups. This indicates that NOX2, and not NOX1, is the main source of O_2_^•^– in this model.

The immunofluorescence studies also provide evidence that NOX2 is an important source of O_2_^•^– generation in this model. Samples from control pups demonstrated relatively minimal co-localization of GP91^phox^ and p47^phox^ on days examined, indicating that p47^phox^ is most likely not activating NOX2 activity in healthy newborn intestines. NEC samples also had little co-localization on D1 and D2. However, there were dramatic increases in GP91^phox^/p47^phox^ co-localization on D4 in NEC pups. This indicates that GP91^phox^ and p47^phox^ are not interacting in control samples or in the first few days of exposure to intestinal injury. However, p47^phox^ localizes to GP91^phox^ on D4, when O_2_^•^– production was the highest. This indicates that NOX2 activity is increased, and a major source of O_2_^•^– in this model.

Prior studies have shown important post-natal changes that are altered in NEC such as barrier function and LPS detoxification [37], [38]. Similarly, we demonstrate that changes in NOX activity occurred with postnatal age. Pups delivered one day prematurely via cesarean delivery had higher NOX activity at birth. This increase in NOX activity could be from prematurity or not undergoing labor, and ultimately requires further investigation. NOX activity rose among control pups during the five days of the study. The increases were significantly different when comparing D0 and D4, and most likely represent physiologic intestinal maturation. In NEC pups, there were two notable changes. The first was from D0-1, when NOX activity decreased. The second and most dramatic differences occurred from D2-3 and D3-4. NOX activity increased several fold during this time. These results imply that repeated exposure to injurious stimuli elevates NOX activity.

These results contribute to the understanding of NEC in several manners. First, this is the first study to associate increased NOX2-dependent O_2_^•^– in NEC. While oxidative stress has been suspected in the disease, our study identifies changes in a known reactive oxygen species source. We also demonstrate that NOX1 and eNOS are not important sources of O_2_^•^– in this model. Another important finding is that NOX activity increases only when the combination of formula feeding, hypoxia, and enteral LPS are present, which demonstrates that multiple factors are required to induce injury in this model, and further underscores the complicated nature of NEC.

There are some limitations in this study. At the time of this study, there was not a NOX2 deficient rat strain. GP91-ds-*tat* was initially designed to prevent the binding of p47^phox^ to GP91^phox^, but may also prevent p47^phox^ from activating NOX1 [39], [40]. However, no studies have clearly demonstrated that NOX1 activity is reduced by GP91-ds-*tat*. NOX1 activation is more efficient with the NOXO1 and NOXA1 subunits than the NOX2 subunits, so the degree of NOX1-dependent O_2_^•^– secondary to p47^phox^ activation is probably minimal. The immunofluorescence studies do not prove that NOX2 is activated on D4 in NEC animals. However, the dramatic differences observed on D4 compared to other time points and experimental conditions indicate that NOX2 activity is elevated.

In closing, this study provides novel evidence that NOX2-dependent O_2_^•^– increases in a rat model of NEC. The results provide evidence that oxidative stress is an important factor in the development and progression of NEC. Inhibition of NOX2 may reduce the incidence and severity of NEC. Future studies will involve the use of genetically modified animals that lack NOX1 or NOX2 to confirm findings from this study.

## Methods

### Ethics Statement

All animal experiments for this study were carried out in strict accordance with the recommendations and approval from the Institutional Animal Care and Use Committee (IACUC) at the Medical College of Wisconsin (Animal Use Application #0092). All members of the research team received appropriate training and were in good standing with the IACUC.

### Animal Model

An *in vivo* NEC model was utilized as previously described [5]. Pregnant female Sprague-Dawley rats (Harlan Laboratories, Madison, WI) were obtained prior to their estimated date of delivery. Control pups were full-term Sprague-Dawley rat pups spontaneously delivered and dam-fed. Experimental pups (NEC pups) were delivered one day prematurely by cesarean section. NEC pups were maintained in an incubator at 37°C and orogastrically fed 0.1–0.25 ml of Esbilac formula, supplemented with Neocate Powdered Formula (Abbott Laboratories, Abbott Park IL) containing LPS (2 mg/kg/day, Sigma-Aldrich, St. Louis, MO), thrice daily when the pups were D0-3. After each feeding, NEC pups were subjected to hypoxia (FiO_2_ of 5% via a hypoxia chamber) for 10 min. Some pups were subjected to only parts of the NEC injury model (formula, formula/hypoxia, or formula/LPS) to clarify contributions from each component. Pups were euthanized on each day of life (D0-D4) to establish a time-sensitive pattern. Pups were euthanized prior to feeding with a ketamine/xylazine injection as per internal IACUC guidelines. One segment was placed in RNAlater (Ambion, Grand Island, NY) and stored at −20°C until isolation. The remaining small intestine was analyzed for NADPH dependent O_2_^•^– production.

### NADPH Dependent O_2_^•^– Production

Intestinal segments were placed in 500 µl of a sucrose-potassium phosphate buffer. Segments were homogenized at 4°C, centrifuged at 13,000 rpm at 4°C for 10 min to remove cell debris, and the supernatant recovered. Protein content was quantified with Bicinconinic acid (BCA) reagent per the manufacturer's instructions (Pierce Chemical Company, Rockford, IL).

Production of O_2_^•^– in small intestines homogenates was quantified as Tiron-inhibitable lucigenin chemiluminescence. Aliquots of 50–300 µg of homogenate were added to wells containing the following reaction components (final concentration): NADPH (100 µM), sucrose (150 mM), and lucigenin (5 µM) (all chemicals from Sigma-Aldrich) [41], [42]. To confirm that NADPH was necessary for O_2_^•^– generation, the cofactor was withheld from the reaction mixture for each sample. Chemiluminescent signals were collected over 30 minutes on a 96 well plate (Bio-Rad), and the total RLU summated (luminometer from Turner Scientific, Madison, WI). Samples from control and NEC groups were analyzed from D0-4. On D4, inhibitors were added to the reaction mixture to quantify contribution from specific enzymes. GP91-ds-*tat* (50 µM, Blood Center of Wisconsin, as previously described [40]) was used to determine the contribution of NOX2 to O_2_^•^– production in homogenates. L-NAME (500 µM, Sigma-Aldrich) was added to determine the contribution of O_2_^•^– production from all NOS isoforms. Background signals were determined by performing the reaction with the respective inhibitors in the absence of an intestinal homogenate, and the resulting chemiluminescence was subtracted from the chemiluminescence observed. The average signal in the presence of Tiron (Sigma-Aldrich) was calculated and subtracted from each sample. Superoxide anion results are expressed as RLU) inhibited by Tiron normalized to intestinal protein (RLU/mg).

### Real-Time PCR (RT-PCR)

RT-PCR for the membrane-bound catalytic site for NOX2, GP91^phox^ (Refseq Accession Number NM_023965.1, primer from Superarray Biosciences, Valencia, CA), NOX1 (Refseq Accession Number NM_053683, primer from IDT, Coralville, IA), and eNOS (Refseq Accession Number NM_021838.2, primer from IDT) were used to quantify time-dependent changes in expression in the terminal ileum. RT-PCR for GAPDH (Refseq Accession Number NM_017008.3, primer from IDT) was used as an internal control. Total RNA was isolated using the Qiagen RNeasyMini Kit, per the manufacturer's protocol. RNA concentration and purity were determined on a NanoDrop spectrophotometer (Molecular Devices, Sunnydale, CA). RNA integrity was measured using the BioRad Experion Analysis Kit; samples with an integrity value of less than 9.0 were excluded (3/53 samples excluded). Complementary DNA (cDNA) was synthesized from 2 µg of DNase-treated total RNA using the iScript cDNA Synthesis kit (Bio-Rad). RT-PCR was performed using ABI Prism 7900HT software (Applied Biosystems, Carlsbad, CA) together with SYBR Green RT-PCR Master Mix (BioRad). All gene-amplification reactions were performed in triplicate. Expression was calculated using the Pfaffl method to determine the fold change in expression in NEC animals relative to control samples from D0 [43].

### Immunofluorescence

Following euthanasia, a 1 cm segment of terminal ileum was embedded in paraffin and cut into 4 micron-thick sections. The sections were deparaffinized at 56°C, immersed in xylene three times and hydrated with ethanol (two times with 100%, two times with 95% and one time with 75% ethanol) for 5 min. For antigen unmasking, slides were heated in 10 mM sodium citrate buffer (pH 6.0) for 15 min prior to treatment with 0.3% hydrogen peroxide for 30 min. The specimens were treated with 5% BSA in TBS-T for 30 min at room temperature followed by overnight incubation with the following primary antibodies, mouse anti-GP91phox, anti-rabbit p-47phox at 4°C, followed with goat anti-rabbit DyLight-649 and goat anti-mouse 488 conjugated 2nd antibody. Images were acquired by confocal microscopy using a Zeiss LSM510.

### Statistical Analysis

A one-way ANOVA was used to determine differences among samples from the same condition for the chemiluminescent experiments. RT-PCR results were calculated from the Pfaffl method, and normalized to control samples from D0 for each respective enzyme. An unpaired t-test was used to compare two specific results for both the chemiluminescent and RT-PCR experiments. Variation was expressed as standard error of the mean (SEM).

## References

[1] Abdullah F, Zhang Y, Camp M, Gabre-Kidan A, Colombani PM (2012) Necrotizing enterocolitis in 20 822 infants: Analysis of medical and surgical treatments. Clinical Pediatrics 49(2):166–171. 10.1177/0009922809349161 20080523

[2] Schnabl KL, Van Aerde JE, Thomson ABR, Clandinin MT (2008) Necrotizing enterocolitis: A multifactorial disease with no cure. World Journal of Gastroenterology 14(14):2142–2161. 18407587 10.3748/wjg.14.2142 PMC2703838

[3] Hsueh W, Caplan MS, Qu X-, Tan X-, De Plaen IG, et al (2003) Neonatal necrotizing enterocolitis: Clinical considerations and pathogenetic concepts. Pediatric and Developmental Pathology 6(1):6–23. 12424605 10.1007/s10024-002-0602-z PMC7098425

[4] Alderton WK, Cooper CE, Knowles RG (2001) Nitric oxide synthases: Structure, function and inhibition. Biochem J 357(3):593–615. 11463332 10.1042/0264-6021:3570593 PMC1221991

[5] Whitehouse JS, Xu H, Shi Y, Noll L, Kaul K, et al (2010) Mesenteric nitric oxide and superoxide production in experimental necrotizing enterocolitis. J Surg Res 161(1):1–8. 19922948 10.1016/j.jss.2009.07.028 PMC3196648

[6] Bedard K, Krause KH (2007) The NOX family of ROS-generating NADPH oxidases: Physiology and pathophysiology. Physiol Rev 87(1):245–313. 17237347 10.1152/physrev.00044.2005

[7] Nauseef WM (2008) Biological roles for the NOX family NADPH oxidases. J Biol Chem 283(25):16961–16965. 18420576 10.1074/jbc.R700045200 PMC2427363

[8] Heymes C, Bendall JK, Ratajczak P, Cave AC, Samuel J-L, et al (2003) Increased myocardial NADPH oxidase activity in human heart failure. J Am Coll Cardiol 41(12):2164–2171. 12821241 10.1016/s0735-1097(03)00471-6

[9] Li J-M, Gall NP, Grieve DJ, Chen M, Shah AM (2002) Activation of NADPH oxidase during progression of cardiac hypertrophy to failure. Hypertension 40(4):477–484. 12364350 10.1161/01.hyp.0000032031.30374.32

[10] Hink U, Li H, Mollnau H, Oelze M, Harmann M, et al (2001) Mechanisms underlying endothelial dysfunction in diabetes mellitus. Circ Res 88(2):E14–22. 11157681 10.1161/01.res.88.2.e14

[11] Lassègue B, San Martín A, Griendling KK (2012) Biochemistry, physiology, and pathophysiology of NADPH oxidases in the cardiovascular system. Circulation Research 110(10):1364–1390. 22581922 10.1161/CIRCRESAHA.111.243972 PMC3365576

[12] Griendling KK, Sorescu D, Ushio-Fukai M (2000) NAD(P)H oxidase: Role in cardiovascular biology and disease. Circ Res 86(5):494–501. 10720409 10.1161/01.res.86.5.494

[13] Lassègue B, Sorescu D, Szöcs K, Yin Q, Akers M, et al (2001) Novel gp91phox homologues in vascular smooth muscle cells: Nox1 mediates angiotensin II-induced superoxide formation and redox-sensitive signaling pathways. Circ Res 88(9):888–894. 11348997 10.1161/hh0901.090299

[14] Arbiser JL, Petros J, Klafter R, Govindajaran B, McLaughlin ER, et al (2002) Reactive oxygen generated by Nox1 triggers the angiogenic switch. Proc Natl Acad Sci U S A 99(2):715–720. 11805326 10.1073/pnas.022630199 PMC117371

[15] Lee MY, Martin AS, Mehta PK, Dikalova AE, Garrido AM, et al (2009) Mechanisms of vascular smooth muscle NADPH oxidase 1 (Nox1) contribution to injury-induced neointimal formation. Arteriosclerosis, Thrombosis, and Vascular Biology 29(4):480–487. 10.1161/ATVBAHA.108.181925 PMC2734189 19150879

[16] Cui X-, Brockman D, Campos B, Myatt L (2006) Expression of NADPH oxidase isoform 1 (Nox1) in human placenta: Involvement in preeclampsia. Placenta 27(4–5):422–431. 15993942 10.1016/j.placenta.2005.04.004 PMC2891430

[17] Yasuda M, Kato S, Yamanaka N, Iimori M, Utsumi D, et al (2012) Potential role of the NADPH oxidase NOX1 in the pathogenesis of 5-fluorouracil-induced intestinal mucositis in mice. American Journal of Physiology - Gastrointestinal and Liver Physiology 302(10):G1133–G1142. 22403796 10.1152/ajpgi.00535.2011

[18] Szanto I, Rubbia-Brandt L, Kiss P, Steger K, Banfi B, et al (2005) Expression of NOX1, a superoxide-generating NADPH oxidase, in colon cancer and inflammatory bowel disease. J Pathol 207(2):164–176. 16086438 10.1002/path.1824

[19] Laurent E, McCoy III JW, Macina RA, Liu W, Cheng G, et al (2008) Nox1 is over-expressed in human colon cancers and correlates with activating mutations in K-ras. International Journal of Cancer 123(1):100–107. 18398843 10.1002/ijc.23423 PMC3774003

[20] Musemeche CA, Caplan M, Hsueh W, Sun X, Kelly A (1991) Experimental necrotizing enterocolitis: The role of polymorphonuclear neutrophils. J Pediatr Surg 26(9):1047–1050. 1941482 10.1016/0022-3468(91)90671-f

[21] Lambeth JD (2004) NOX enzymes and the biology of reactive oxygen. Nat Rev Immunol 4(3):181–9. 15039755 10.1038/nri1312

[22] Piccoli C, Ria R, Scrima R, Cela O, D'Aprile A, et al (2005) Characterization of mitochondrial and extra-mitochondrial oxygen consuming reactions in human hematopoietic stem cells. Novel evidence of the occurrence of NAD(P)H oxidase activity. J Biol Chem 15 280(28):26467–76 Epub 2005 May 9. 10.1074/jbc.M500047200 15883163

[23] Hilburger EW, Conte EJ, McGee DW, Tammariello SP (2005) Localization of NADPH oxidase subunits in neonatal sympathetic neurons. Neurosci Lett. 22 377(1):16–9. 10.1016/j.neulet.2004.11.066 15722179

[24] Perrone S, Tataranno ML, Negro S, Longini M, Marzocchi B, et al (2010) Early identification of the risk for free radical-related diseases in preterm newborns. Early Hum Dev 86(4):241–244. 20466493 10.1016/j.earlhumdev.2010.03.008

[25] Perrone S, Tataranno ML, Negro S, Cornacchione S, Longini M, et al (2012) May oxidative stress biomarkers in cord blood predict the occurrence of necrotizing enterocolitis in preterm infants? Journal of Maternal-Fetal and Neonatal Medicine 25 (Suppl. 1)128–131. 22339378 10.3109/14767058.2012.663197

[26] Aydemir C, Dilli D, Uras N, Ulu HO, Oguz SS, et al (2011) Total oxidant status and oxidative stress are increased in infants with necrotizing enterocolitis. J Pediatr Surg 46(11):2096–2100. 22075338 10.1016/j.jpedsurg.2011.06.032

[27] Clark DA, Fornabaio DM, McNeill H, Mullane KM, Caravella SJ, Miller MJS (1988) Contribution of oxygen-derived free radicals to experimental necrotizing enterocolitis. Am J Pathol 130(3):537–542. 3348358 PMC1880686

[28] Baregamian N, Song J, Jeschke MG, Evers BM, Chung DH (2006) IGF-1 protects intestinal epithelial cells from oxidative stress-induced apoptosis. J Surg Res 136(1):31–37. 16999977 10.1016/j.jss.2006.04.028 PMC2613687

[29] Baregamian N, Song J, Bailey CE, Papaconstantinou J, Evers BM, et al (2009) Tumor necrosis factor-a and apoptosis signal-regulating kinase 1 control reactive oxygen species release, mitochondrial autophagy and c-jun N-terminal kinase/p38 phosphorylation during necrotizing enterocolitis. Oxidative Medicine and Cellular Longevity 2(5):297–306. 20716917 10.4161/oxim.2.5.9541 PMC2835918

[30] Kelly N, Friend K, Boyle P, Zhang XR, Wong C, et al (2004) The role of the glutathione antioxidant system in gut barrier failure in a rodent model of experimental necrotizing enterocolitis. Surgery 136(3):557–566. 15349102 10.1016/j.surg.2004.05.034

[31] Babior BM (2004) NADPH oxidase. Curr Opin Immunol 16(1):42–47. 14734109 10.1016/j.coi.2003.12.001

[32] Rentea RM, Welak SR, Fredrich K, Donohoe D, Pritchard K, et al (2013) Early enteral stressors in newborns increase inflammatory cytokine expression in a neonatal necrotizing enterocolitis rat model. European Journal of Pediatric Surgery 23(1):39–47. 23165517 10.1055/s-0032-1329704 PMC5664148

[33] Nadler EP, Dickinson E, Knisely A, Zhang X-R, Boyle P, et al (2000) Expression of inducible nitric oxide synthase and interleukin-12 in experimental necrotizing enterocolitis. J Surg Res 92(1):71–77. 10864485 10.1006/jsre.2000.5877

[34] Upperman JS, Potoka D, Grishin A, Hackam D, Zamora R, et al (2005) Mechanisms of nitric oxide-mediated intestinal barrier failure in necrotizing enterocolitis. Semin Pediatr Surg 14(3):159–166. 16084403 10.1053/j.sempedsurg.2005.05.004

[35] Xia Y, Zweier JL (1997) Superoxide and peroxynitrite generation from inducible nitric oxide synthase in macrophages. Proc Natl Acad Sci USA 94(13):6954–6958. 9192673 10.1073/pnas.94.13.6954 PMC21266

[36] Xia Y, Roman LJ, Masters BSS, Zweier JL (1998) Inducible nitric-oxide synthase generates superoxide from the reductase domain. J Biol Chem 273(35):22635–22639. 9712892 10.1074/jbc.273.35.22635

[37] Rentea RM, Liedel JL, Welak SR, Cassidy LD, Mayer AN, et al (2012) Intestinal alkaline phosphatase administration in newborns is protective of gut barrier function in a neonatal necrotizing enterocolitis rat model. J Pediatr Surg 47(6):1135–1142. 22703783 10.1016/j.jpedsurg.2012.03.018

[38] Heinzerling NP, Liedel JL, Welak SR, Fredrich K, Biesterveld BE, et al (2014) Intestinal alkaline phosphatase is protective to the preterm rat pup intestine. J Pediatr Surg 49(6):954–960. 24888842 10.1016/j.jpedsurg.2014.01.031 PMC4130394

[39] Yang M, Kahn AM (2006) Insulin-stimulated NADH/NAD+ redox state increases NAD(P)H oxidase activity in cultured rat vascular smooth muscle cells. Am J Hypertens 19(6):587–92. 16733230 10.1016/j.amjhyper.2005.11.017

[40] Rey FE, Cifuentes ME, Kiarash A, Quinn MT, Pagano PJ (2001) Novel competitive inhibitor of NAD(P)H oxidase assembly attenuates vascular O2 - and systolic blood pressure in mice. Circ Res 89(5):408–414. 11532901 10.1161/hh1701.096037

[41] Griendling KK, Minieri CA, Ollerenshaw JD, Alexander RW (1994) Angiotensin II stimulates NADH and NADPH oxidase activity in cultured vascular smooth muscle cells. Circulation Research 74(6):1141–1148. 8187280 10.1161/01.res.74.6.1141

[42] Teng R-, Eis A, Bakhutashvili I, Arul N, Konduri GG (2009) Increased superoxide production contributes to the impaired angiogenesis of fetal pulmonary arteries with in utero pulmonary hypertension. American Journal of Physiology - Lung Cellular and Molecular Physiology 297(1):L184–L195. 19429773 10.1152/ajplung.90455.2008 PMC2711810

[43] Pfaffl MW (2001) A new mathematical model for relative quantification in real-time RT-PCR. Nucleic Acids Res 29(9). 10.1093/nar/29.9.e45 PMC55695 11328886

